# HmbC, a Protein of the HMG Family, Participates in the Regulation of Carotenoid Biosynthesis in *Fusarium fujikuroi*

**DOI:** 10.3390/genes14081661

**Published:** 2023-08-21

**Authors:** Marta Franco-Losilla, Steffen Nordzieke, Ingo Feldmann, M. Carmen Limón, Javier Avalos

**Affiliations:** 1 Departamento de Genética, Facultad de Biología, Universidad de Sevilla, 41012 Sevilla, Spain; martafranco@us.es (M.F.-L.); avalos@us.es (J.A.); 2 Leibniz-Institut für Analytische Wissenschaften—ISAS—e.V., 44227 Dortmund, Germany; feldmann@isas.de

**Keywords:** high-mobility group, HmbC protein, carotenoids, pull-down assay, *carS* gene, protoplast formation

## Abstract

In the fungus *Fusarium fujikuroi*, carotenoid production is up-regulated by light and down-regulated by the CarS RING finger protein, which modulates the mRNA levels of carotenoid pathway genes (*car* genes). To identify new potential regulators of *car* genes, we used a biotin-mediated pull-down procedure to detect proteins capable of binding to their promoters. We focused our attention on one of the proteins found in the screening, belonging to the High-Mobility Group (HMG) family that was named HmbC. The deletion of the *hmbC* gene resulted in increased carotenoid production due to higher mRNA levels of *car* biosynthetic genes. In addition, the deletion resulted in reduced *carS* mRNA levels, which could also explain the partial deregulation of the carotenoid pathway. The mutants exhibited other phenotypic traits, such as alterations in development under certain stress conditions, or reduced sensitivity to cell wall degrading enzymes, revealed by less efficient protoplast formation, indicating that HmbC is also involved in other cellular processes. In conclusion, we identified a protein of the HMG family that participates in the regulation of carotenoid biosynthesis. This is probably achieved through an epigenetic mechanism related to chromatin structure, as is frequent in this class of proteins.

## 1. Introduction

High-mobility-group (HMG) proteins are a class of non-histone proteins that play regulatory roles in chromatin structure and influence its higher order organization [1,2]. As an active part of chromatin architecture, these proteins modulate nucleosome DNA accessibility through their influence on its higher order and are involved in the control of chromatin remodeling, transcription, DNA replication, and DNA repair. The alternation between the different accessibility states is controlled by chromatin remodeling complexes, which affect the strength of DNA-histone interactions through DNA methylation, histone post-translational modification, or access by specific transcription factor or other proteins [3].

There are three major families of HMG proteins: HMGA (HMG AT Hook), HMGB (HMG box), and HMGN (HMG nucleosome binding) [4]. Each HMG protein contains one or more DNA- or nucleosome-binding domains that define its mechanism of interaction with chromatin, usually accompanied by regulatory domains [2]. HMGB family proteins typically contain two HMG boxes responsible for DNA interaction, followed by a C-terminal acidic tail mediating specific interaction features. The HMGB box allows for non-sequence specific binding to DNA, although these proteins bind with high affinity to already bent or distorted DNA structures [4,5].

Studies addressed to elucidate the role of HMGB proteins in transcription in different biological systems revealed diverse mechanisms of action. One of them is the facilitation of binding of chromatin remodeling proteins [6]. However, a role as transcriptional repressors through interaction with TATA-binding protein (TBP), preventing TBP binding on promoters, was described for the human HMGB1 protein [7]. In another mechanism, known as “hit-and-run”, the HMGB protein promotes the stable interaction of other transcription factors with their DNA binding sites, after which HMGB dissociates from the resulting complex and leaves the factors stably bound to DNA [1]. Although there are HMGB proteins that bind to specific sequences, they frequently lack such specificity and are abundantly distributed chromatin-associated proteins, responsible for facilitating the binding of regulatory proteins by modulating the local chromatin environment and binding nucleosomes at entry/exit points [8,9].

Chromatin structure has an emerging role in the regulation of fungal secondary metabolism, including *Fusarium* species [10]. Fungi of the genus *Fusarium* are widely used research models, mainly because they are the causative agents of numerous pathologies, especially in plants [11]. Their pathogenic effects are combined with their enormous capacity to produce secondary metabolites, some with biotechnological applications but others as possible harmful agents for human and animal health [12]. A representative species is *F. fujikuroi*, a rice pathogen known for its ability to produce gibberellins, widespread plant hormones produced also by some bacteria and fungi [13]. This is just one of the many metabolites produced by these fungi. Genomic analysis revealed an enormous potential of this species for secondary metabolite production [14,15].

Among the different metabolites synthesized by *Fusarium* species are carotenoids, fat-soluble terpenoid pigments produced by photosynthetic species and numerous bacteria and fungi, which are obtained by animals through dietary intake [16]. Due to its easy traceability and biotechnological potential, the synthesis of carotenoids has received special attention in *F. fujikuroi*, which is currently one of the reference models in the synthesis of these pigments in fungi [16]. All the biosynthetic genes of the *Fusarium* carotenoid pathway, known as *car* genes, have been elucidated, and their availability has facilitated studies on their regulation, involving different environmental agents, especially light [16]. At least two major regulatory proteins are involved in the control of their expression, a positive regulator, the White collar 1-like protein WcoA, which mediates the response to light [17], and a negative regulator, the RING-finger protein CarS [18], which maintains synthesis at moderate levels. Loss of function of *carS* gene results in transcriptional deregulation of the structural *car* genes [16], with intense pigmentation due to overaccumulation of carotenoids, while *carS* overexpression results in inhibition of the pathway and an albino phenotype [19]. In both cases, there is a correlation between carotenoid content and mRNA levels of the corresponding *car* genes.

Here, we described the identification of a novel regulatory protein involved in carotenoid biosynthesis, the HMG protein HmbC, found in a screening of proteins bound to *car* genes promoters by a pull-down strategy. Deletion of *hmbC* gene resulted in a partial deregulation of the carotenoid pathway, as well as changes in sensitivity to cell wall degrading enzymes or minor developmental alterations under stress conditions.

## 2. Materials and Methods

### 2.1. Strains and Culture Conditions

The wild-type strain *F. fujikuroi* IMI58289 was obtained from the Imperial Mycological Institute (Kew, Surrey, England). SG39 is a carotenoid overproducing strain with a mutation in the regulatory gene *carS* [18]. Δ*hmbC* SG323 and SG324 and ectopic SG322 transformants were obtained in this work. *Saccharomyces cerevisiae* strain FY834 (*MATα*, *ura3-52*, *leu2*∆*1*, *trp1*∆*63*, *his3*∆*200*, *lys2*∆*202*) was used for plasmid constructions by homologous recombination.

The strains were grown in liquid or solidified with 16 g/L agar DG minimal medium [20] or DGasn medium, the same as DG but containing asparagine instead of NaNO_3_. In the case of stress studies, the media were supplemented with the chemicals indicated in results. For phenotypic and molecular analysis, strains were cultured at 30 °C for 3–7 days depending on the assay and under light or dark conditions. For sporulation, strains were grown in EG agar medium [20] for 7 days under light at 24 °C. For expression analysis, 100 mL of DG medium in 500 mL flasks were inoculated with 10^6^ spores of the corresponding strain and incubated for 3 days in an orbital shaker at 150 rpm in dark, and 25 mL of mycelium samples were collected, filtrated, and frozen at −80 °C. To obtain protein extracts for the pull-down assays, wild type and SG39 *carS* mutant were grown for 3 days in the dark in flasks with DG medium. For carotenoid analyses, Petri dishes with DG were inoculated at seven symmetrically distributed points of the required strain and incubated at 30 °C for 7 days under light or dark conditions. For analyses in submerged cultures, strains were grown for 7 days, the mycelium was collected by filtration, and frozen at −80 °C. Carotenoids were extracted with acetone from lyophilized mycelia following a standard protocol [20].

Light conditions consisted in the exposure to four fluorescent tubes (Philips TL-D 18W/840) at ca. 0.6 m, providing at this distance a light intensity of 7 W m^−2^.

### 2.2. DNA Isolation and PCR Assays

Genomic DNA was obtained with the commercial kit NucleoSpin Plant II (Macherey-Nagel GmbH & Co. KG, Düren, Germany). For PCR assays, two different DNA polymerases were used. BIOTAQ^TM^ DNA polymerase (Bioline GmbH, Luckenwalde, Germany) was used for standard reactions. Because of its lower mutation rate, Q5 High-Fidelity DNA Polymerase (New England BioLabs, Ipswich, MA, USA) was used for cloning, sequencing purposes, or overlap extension PCR [21]. The primer sets (PS) are described in Supplementary Material, Table S1.

### 2.3. Plasmid Construction and Transformation

To generate Δ*hmbC* mutants, a plasmid containing a hygromycin-resistance cassette (Hyg^R^) surrounded by the 5′ and 3′ fragments of the *hmbC* gene was obtained in several steps that included homologous recombination with plasmid pRS426 and fusion PCR (Supplementary Material, Figure S1). Step 1: a 1 kb fragment containing the 5′ region of *hmbC* gene and a 1 kb fragment containing the 3′ region of *hmbC* gene were amplified from *F. fujikuroi* genomic DNA by PCR with primer sets PS1 and PS2, respectively. Both fragments contained tails to allow recombination with the multicloning site of pRS426 plasmid and the Hyg^R^ cassette, which was amplified in parallel by PCR from plasmid pSCN44 with primer set PS4. Step 2: The three PCR fragments were introduced in *S. cerevisiae* FY834 with linearized plasmid pRS426 to obtain the final plasmid. However, only the plasmid with 5′ *hmbC* and Hyg^R^ cassette could be identified in the yeast transformants. This made necessary an additional step. Step 3: A fragment containing the 5′ *hmbC* region and the Hyg^R^ cassette was obtained and amplified with PS3 primer set from the plasmid obtained in step 2. The resulting 5′ hmbC–Hyg^R^ fragment was joined to the 3′ *hmbC* region by fusion PCR using primer set PS5. The resulting *hmbC*-deletion cassette was finally ligated in pSPARK vector, to give pMFL11 plasmid.

To generate Δ*hmbC* mutants, protoplasts obtained from the wild type IMI58289 were transformed with the deletion cassette obtained by PCR from pMFL11, as previously described [20].

### 2.4. Southern Blot

The probe for the Southern blot was obtained by PCR using PS8 primer set, PCR DIG DNA Labeling mix (Roche, Mannheim, Germany), and BIOTAQ^TM^ DNA polymerase (Bioline GmbH) and polymerase reaction buffer. Ten μL of 200 μM PCR DIG labeling, containing unlabeled dNTPs and digoxigenin-11-dUTP, were added to a 100 μL PCR reaction performed following the instructions of BIOTAQ^TM^ DNA polymerase, with annealing temperatures adjusted according to used primers. The 804 pb probe was purified using the ISOLATE II PCR and Gel kit (Bioline GmbH).

For the Southern blot, 2–4 μg of genomic DNA were digested overnight with the indicated restriction enzyme, loaded in 0.8% agarose gel, separated by electrophoresis with DIG-labeled DNA Molecular Weight Marker VII (Roche), and subjected to the protocol previously described [22], using a positive charged nylon membrane (Hybond-N, Amersham, GE Healthcare, Chicago, IL, USA). The membrane was preincubated with “DIG Easy Hyb™ Granules (Roche) buffer solution for 1 h in a glass cylinder in a hybridization oven (HB-100 Hybridizer, UVP) at 42 °C. Afterwards, 35 ng/mL of DIG-labelled probe were added in a fresh buffer solution and incubated overnight at the same temperature. This was followed by two washes with 2X SSC + 0.1% SDS at room temperature for 5 min and two washes with 2X SSC + 0.1% SDS at 68 °C for 15 min. The membrane was equilibrated with maleic buffer and incubated in Blocking Reagent solution (Roche) for 1 h. Then, fresh stock of blocking solution with a 1:10,000 dilution of anti-DIG alkaline-phosphatase antibody (Merck KGaA, Darmstadt, Germany) was added for 30 min. The membrane was washed twice for 15 min with maleic buffer + 0.3% Tween 20, exposed to CDP-Star^®^ ready to use (Roche), and signals were detected in an Odyssey Fc Imaging System (LI-COR, Lincoln, NE, USA).

### 2.5. Expression Analyses

Frozen samples were ground with a FAST-PREP24 (MP Biomedicals, LLC, Irvine, CA, USA) disruption system and RNA extraction was achieved with the commercial RNeasy^®^ Plant Mini kit (QIAGEN, Hilden, Germany). Retrotranscription to cDNA was performed with 2.5 μg, using the Transcriptor First Strand cDNA synthesis kit (Roche). Final cDNA concentrations were adjusted to 25 ng/μL. RT-PCR was performed with a LightCycler 480 real-time instrument (Roche), using the LightCycler 480 SYBR green I Master kit (Roche). The primer sets used for the amplification and detection or the mRNA of *carB*, *carRA*, and *carS* genes are listed in Supplementary Material, Table S2, as well as those used for the detection of the β1-tubulin gene, *FFUJ_04397*, and the glyceraldehyde-3-phosphate dehydrogenase (*gpdA*) gene, *FFUJ_13490*, used to normalize the transcript levels of the investigated genes. The data were relativized to the value of the wild-type strain grown in the dark.

### 2.6. Nuclei Extraction

Mycelia were separated from the medium using porcelain filters, the resulting mycelial pads were washed with 0.96% NaCl solution and stored at −80 °C until use. For extraction, each pad was kept frozen in liquid nitrogen in a mortar and homogenized using a pestle. The broken sample was distributed in two 15 mL Falcon tubes containing 6 mL of buffer A (1 M sorbitol, 7% *w/v* Ficoll, 20% glycerol, 50 mM Tris-HCl pH 7.5, 5 mM MgAc, 5 mM EGTA, 3 mM CaCl_2_, 0.2% *w/v* Protease Inhibitor Cocktail IV, 2 µM leupeptin, and 0.5 mM DTT). The resuspended extract was filtered through a syringe filled with sterile cloth into a beaker glass containing a magnetic stir bar. During stirring, 2 volumes of buffer B (10% glycerol, 25 mM Tris-HCl pH 7.5, 5 mM MgAc, 5 mM EGTA, 0.2% *w/v* Protease Inhibitor Cocktail IV, 2 µM leupeptin, 1 mM DTT) were added slowly to the solution. Diluted homogenate was layered over 15 mL of a solution consisting of a 5.55 mL of buffer A and 9.45 mL of buffer B (1:1.7 mix) in a 50 mL Falcon tube. The mixture was centrifuged at 4200 rpm for 10 min at 4 °C to remove cell debris, and supernatant was collected and layered slowly onto a 5 mL sucrose gradient (1 M sucrose, 10% glycerol, 25 mM Tris pH 7.5, 5 mM MgAc). After a second centrifugation step at 7500 rpm for 30 min at 4 °C, the supernatant, corresponding to the nuclear-free cytoplasmic fraction, was collected, whereas the nuclear pellet was resuspended in ice-cold storage buffer (25% glycerol, 5 mM MgAc, 0.1 mM EDTA, 20 mM Tris-HCl pH 7.5, 0.2% *w/v* protease inhibitor cocktail IV, 2 µM leupeptin, 3 mM DTT). Nuclei were broken down using 0.5% Nonidet-P40 and nucleic extract was used in streptavidin pull-down.

### 2.7. Full Protein Extraction, Streptavidin Pull-Down, and Precipitation

To extract proteins, mortar-ground frozen mycelium was mixed with protein extraction buffer (100 mM Tris, 250 mM NaCl, 2 mM EDTA, 10% glycerol, 0.5% NP-40, 1% 100 mM PMSF, 0.2% proteinase inhibitor Cocktail IV, 0.2% 1 M DTT) and centrifuged for 40 min at 12,000 rpm at 4 °C. Afterwards, the supernatant was transferred to reaction tubes and concentration was determined using Bradford solution.

For the pulldown assays, DNA fragments of *carB* and *carRA* promoters were obtained by PCR using specific biotin-labelled forward primers in combination with non-labelled reverse primers, with expected sizes of 548 bp (P*carB*) and 834 bp (P*carRA*/carX), respectively. Primers and setups for these PCRs are described in Supplementary Material, Tables S3 and S4, respectively.

Purification of proteins by streptavidin was performed according to the manufacturer’s instructions with the following setup: binding of biotinylated probe DNA was performed using 200 µL of 400 ng/µL probe, either derived directly from PCR through sodium acetate precipitation or by means of extraction from agarose gels using the Isolate II PCR and Gel kit (Meridian Bioscience, Cincinnati, OH, USA). Incubation with cell lysate was performed using 750 µL of nucleic extract and cleared protein lysate, respectively, supplemented with 50 µg of salmon sperm DNA. Elution was performed using 10 mM EDTA in 95% formamide and incubating at 65 °C for 5 min.

For protein precipitation, 300 µL of 100% trichloroacetic acid were added per mL of the eluate derived from the streptavidin pull-down. The mixture was incubated in ice for 30 min, with vigorous shaking every 5 min in a vortex. After centrifugation for 30 min at 15,000 rpm at 4 °C, supernatant was discarded leaving a small rest to ensure that no pellet was lost. The pellet was washed two times with ice cold acetone and dried using a speed vac microcentrifuge.

### 2.8. Mass Spectrometry

Sample pellets were resuspended in 10 µL of 2 M GuHCl (guadinine hydrochloride) followed by the addition of 90 µL of ABC buffer and several ultrasonication steps in between. Protein amount was determined using a NanoDrop 2000 spectrophotometer (Thermo Scientific, Dreieich, Germany). After reduction in dithiothreitol (DTT) and alkylation with iodoacetamide (IAA) to stabilize disulfide bonds, the sample was digested with trypsin overnight at 37 °C using a protein to trypsin ratio of 1:20.

Samples were analyzed in a Q Exactive HF mass spectrometer coupled to an U3000 nano RSLC HPLC, both instruments from Thermo Scientific. All solvents were ultra-liquid chromatography (UCL)-mass spectrometry (MS) grade (Biosolve, Valkenswaard, Netherlands). Samples were loaded in 0.1% trifluoroacetic acid (TFA) at a flow rate of 20 μL/min onto a trap column (Acclaim^TM^ PepMap ^TM^ C18, 0.1 × 20 mm, 5 μm, 100 Å) for online SPE. After 5 min, the trap column was switched in line with the main column. The samples were separated on a 50 cm column (Acclaim^TM^ PepMap^TM^ C18, 0.075 × 500 mm, 2 μm, 100 Å) with a flow rate of 250 nL/min at 60 °C. Peptides were separated using a binary acetonitrile (ACN) gradient in the presence of 0.1% formic acid with 5 min isocratic part at the beginning followed by a linear gradient with increasing acetonitrile from 2.5% to 35% B in 52 min.

The Q Exactive HF mass spectrometer was operated in data-dependent acquisition (DDA) mode with survey scans acquired at a resolution of 60,000 followed by maximal 15 tandem mass spectrometry (MS/MS) scans at a resolution of 15,000 (top 15). Precursor ions were selected for MS/MS by intensity, isolated in a 1.6 *m/z* window, and subjected to fragmentation by higher-energy-collision-induced dissociation using a normalized collision energy (NCE) of 27. Automatic gain control (AGC) target values were set to 10^6^ and 5 × 10^4^, and the maximum ion injection was set to 120 ms for MS and to 250 ms for MS/MS. Dynamic exclusion was set to 12 s, and the polysiloxane at *m/z* 371.1012 was used as an internal calibrant.

Data were evaluated using Proteome Discoverer 1.4 software (Thermo Scientific, Dreieich, Germany) using maximum missed cleavage sites of 2, peptide cut off score of 5, a precursor mass tolerance of 5 ppm, and a fragment mass tolerance of 0.02 Da. Oxidation of methionine was selected as dynamic modification and carbamidomethylation of cysteine as static modification.

### 2.9. Bioinformatic and Statistical Analysis

DNA sequences and genome information were obtained through the National Centre of Biotechnology and Informatics (NCBI) (https://www.ncbi.nlm.nih.gov, accessed on 18 August 2023), the Ensemble Fungi server (http://fungi.ensembl.org/index.html, accessed on 18 August 2023), and FungiDB (https://fungidb.org/fungidb/app, accessed on 18 August 2023). The BLAST and BLASTP analyses were performed through the NCBI server. DNA sequencing data were analyses with Clustal Omega sequence alignment software from European Bioinformatics Institute of the European Molecular Biology Laboratory, EMBL-EBI (https://www.ebi.ac.uk/Tools/msa/clustalo/, accessed on 18 August 2023).

Unpaired Student’s t test was used to analyze statistical significance of differences in pairwise data comparisons using GraphPad Prism 8. Levels of significance are indicated with asterisks: * *p* < 0.05, ** *p* < 0.01, *** *p* < 0.001, **** *p* < 0.0001. Differences were not considered statistically significant for *p* values ≥ 0.05.

## 3. Results

### 3.1. Identification of Proteins Binding to car Promoters by Pull-Down Assay

A pull-down assay was performed with the aim of identifying specific transcription factors binding to the regulatory regions of the *carB* and *carRA*/*carX* genes (intergenic region of the divergently transcribed genes *carRA* and *carX*, Supplementary Material, Figure S2A), with key roles in carotenoid biosynthesis (Supplementary Material, Figure S2B). For this goal, promoter fragments were obtained by PCR using specific biotin-labeled forward primers in combination with non-labeled reverse primers, with expected sizes of 548 bp (P*carB*) and 834 bp (P*carRA*/*carX*). Setups for these PCRs are described in Supplementary Material, Table S4. The resulting biotinylated promoter fragments were visualized on gel electrophoresis (Supplementary Material, Figure S2C), coupled separately to streptavidin-beads, and incubated consecutively with nucleic extracts and full protein extracts from either *F*. *fujikuroi* wild type or SG39 *carS* mutant. As controls, non-biotinylated DNA from herring spermatozoa was used as bait and pure protein extraction buffer without protein as prey. Binding of biotinylated DNA probes to streptavidin beads was checked on various steps of the streptavidin pull-down process (Supplementary Material, Figure S2D). Free-biotinylated DNA was enriched in S2 over S1, showing the ongoing saturation of biotin-contact sites of the beads. The washing step contained only a minor fraction of excess biotin-P*carB* (W1), whereas a large fraction of biotin-P*carB* was eluted by incubation at 75 °C for 15 min (ELU). The process was repeated with the same results for P*carRA*/*carX*.

Final eluates were precipitated with trichloroacetic acid and stored at −70 °C until mass spectrometry analysis. Masses were mapped against proteome of *F. fujikuroi* IMI58289 and individual peptides were assigned. After subtracting the hits of the water control, 449 different proteins were assigned by individual peptides in all eight experiments (two repetitions for each of the four samples). Results varied in quality between baits used: whereas P*carB* detected a mean of 192 different proteins, this number was reduced to a mean of 53 using P*carRA*/*carX*, whereas the type of lysed protein extracts showed no difference. The most abundant proteins in the analysis are described in Table 1, which shows a diverse list of proteins, many of them with functions presumably related to DNA processes. Although it was only found bound to the *carB* promoter, CCT62893 protein, orthologous of the Hmo1p HMG protein of *S. cerevisiae*, caught our attention.

### 3.2. Proteins with HMG Domains in F. fujikuroi

To place CCT62893 protein in context, a survey of HMG proteins in the proteome of *F. fujikuroi* was performed by searching for those containing the “High mobility group box superfamily” domain (IPR036910) in the EnsembleFungi database. Nine proteins were identified, in addition to CCT62893 (Table 2). The predicted HMG proteins of *F. fujikuroi* were frequently accompanied by diverse additional domains and had a large variability in size, ranging from 100 residues of CCT62544 to over 1000 residues of CCT66004. One of them was CCT62089, which was very abundant in the pull-down assay. None of the other putative HMG proteins were found in the screening.

**Figure 1:**
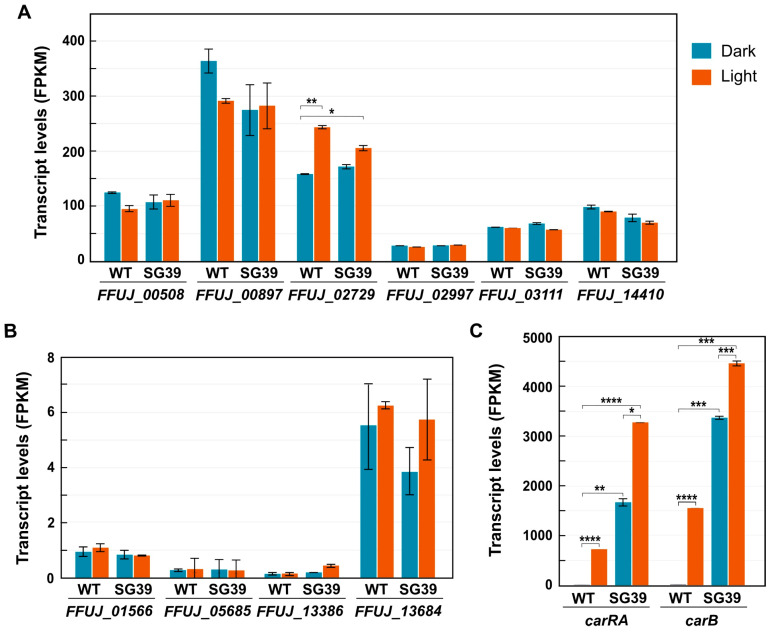
Transcript levels (FPKM) of the genes encoding the proteins with HMG domains described in Table 1. (**A**) Expression of genes with relatively high transcription levels. (**B**) Genes with low transcription levels. (**C**) Data for the genes *carRA* and *carB* are included as examples of strong activations by light and by the *carS* mutation. WT: Wild type, SG39: *carS* mutant. Red bars: one h illumination. Data retrieved from the datasets of a formerly realized RNA-Seq study on the effects of light and CarS protein on the *F. fujikuroi* transcriptome [23]. Only statistically significant differences, according to Student’s *t* test, are indicated: * *p <* 0.05, ** *p* < 0.01, *** *p* < 0.001, **** *p* < 0.0001.

Expression data for the 10 genes were obtained from previous RNA-Seq datasets of our group on the effect of light or *carS* mutation on the *F. fujikuroi* transcriptome [23]. The data showed that the encoding genes also exhibited a high variability in their expression patterns (Figure 1). Transcript levels were hardly detectable in three of the genes (*FFUJ_01566*, *_05685* and *_13386*), one of which was the *mat1-2-1* gene (*FFUJ_05685*), while the others exhibited diverse expression levels. Except for a possible minor light induction in the case of *FFUJ_02729*, none of the genes were importantly affected by light or by the CarS protein involved in the regulation of carotenogenesis. This can be appreciated when compared to the severe effects of light and *carS* mutation on the mRNA levels of the structural genes of the carotenoid pathway *carRA* and *carB*, on whose promoters the pull-down assay was carried out. 

### 3.3. Features of hmbC Gene and HmbC Protein

We focused our attention on *FFUJ_00508* gene, encoding the predicted nonhistone chromosomal CCT62893 protein of the HMG-box family. Two orthologs of *FFUJ_00508* have been investigated in other fungi: HmbC in *Aspergillus nidulans* [24] and Hmo1 in *S. cerevisiae* [25,26,27]. A phylogenetic tree of the 10 HMG proteins identified in *F. fujikuroi* with these two proteins showed a closest relation of HmbC with its *A. nidulans* and *S. cerevisiae* counterparts than with any of the other *F. fujikuroi* HMG proteins (Supplementary Material, Figure S3). The relationship of CCT62893 with these proteins is very evident with the one of *A. nidulans*, as revealed the Clustal alignments, that drew our attention especially to the high conservation of the A box of the HMG region, which was about 50% identical between *F. fujikuroi* and *A. nidulans*, while it was of only 30% in the case of *S. cerevisiae* (Supplementary Material, Figure S4). However, the rest of the proteins showed lower conservation, suggesting functional divergences of these proteins in the different fungal lineages. Because of the lower taxonomic distance with *A. nidulans*, following our usual genetic terminology, we will refer to *FFUJ_00508* hereafter as *hmbC* and CCT62893 as HmbC. The *F. fujikuroi hmbC* gene is localized on chromosome 1 and spans 1548 bp from start codon to stop codon. In the genome annotation in the EnsembleFungi database, the gene contains two predicted introns, expected to result in a mature mRNA containing an ORF of 1065 nucleotides whose translation would give rise to a 354-residue protein.

### 3.4. Deletion of hmbC

To determine the function of HmbC in *F. fujikuroi*, deletion mutants were generated from the wild-type strain by replacing its *hmbC* sequence with a hygromycin resistance cassette (Hyg^R^). For this goal, pMFL11 vector was constructed, in which the *hmbC* sequence was replaced by the Hyg^R^ cassette surrounded by approximately 1 kb of the 5′ and 3′ *hmbC* flanking regions. This construct was incubated with wild-type protoplasts and transformants resistant to hygromycin B were selected. Nine potential transformants were genetically purified by isolation of uninucleate microconidia in three successive steps. Finally, molecular analyses were performed to identify those containing the expected *hmbC* replacement (Supplementary Material, Figure S5A). Different PCRs were carried out in comparison to the wild type (Supplementary Material, Figure S5B). The first PCR using an internal *hph* primer and another one from the 5′ upstream *hmbC* region, absent in pMFL11 plasmid, confirmed the introduction of the Hyg^R^ sequence in the *hmbC locus* in eight of the nine transformants. This introduction must have occurred by recombination through the homologous *F. fujikuroi* sequences surrounding the Hyg^R^ cassette. Interestingly, the eight transformants displayed two different phenotypes (Supplementary Material, Figure S5C). Transformants 4, 24, and 34 showed a wild-type appearance, but transformants 1, 17, 20, 23, and 31 exhibited more intense orange pigmentation.

Homologous recombination can result in single integrations or gene substitutions by double recombination events. To distinguish between the two possibilities, a second PCR was carried out using primers that bind to the surrounding region to be substituted. The replaced genomic DNA fragment has about 300 bases less than the Hyg^R^ cassette, so that in case of replacement in the *F. fujikuroi* genome, a detectable shift of the PCR product should occur. Such a shift was observed in all but one transformant, which showed a wild-type pigmentation (Supplementary Material, Figure S5D). However, the wild-type phenotype of two of the seven positive transformants had no obvious explanation. Such amplification could also be obtained if other integrations of the cassette and its surrounding genomic DNA had occurred. To confirm this, Southern blot hybridizations of DNA from selected transformants were performed to verify the loss of the *hmbC* gene. We chose three transformants with altered pigmentation, 1, 17, and 31, and a wild-type-pigmented transformant, 34. For the hybridization, a probe from the *hmbC* 3′ region present in the deletion cassette (Supplementary Material, Figure S6A) was used to provide information not only on the presence of the intact *hmbC* region in the transformants but also on the number of copies of the sequence introduced in the transformation process.

The Southern blot result revealed a single band of the expected size in the wild type with each of the two restriction enzymes used (Supplementary Material, Figure S6B). This band disappeared in the deep-pigmented transformants 1, 17, and very probably also in 31, but not in the transformant 34, which, in the *Pst*I digestion, displayed a clear additional band, consistent with an ectopic integration (Supplementary Material, Figure S6C). Additional bands were also observed in the other transformants, indicating that multiple integrations are not rare. In conclusion, the hybridization results confirmed the *hmgbC* replacement by the Hyg^R^ cassette in transformants 1, 17, and 31 and at least an additional ectopic integration of the deletion cassette. Hereafter, the Δ*hmbC* transformants will be referred to as SG323 (1), SG324 (17), and SG325 (31). The transformant 34, retaining the *hmbC* gene, will be eventually used as an additional negative control and will be referred to as SG322.

### 3.5. Effect of ΔhmbC Deletion on Carotenogenesis

**Figure 2:**
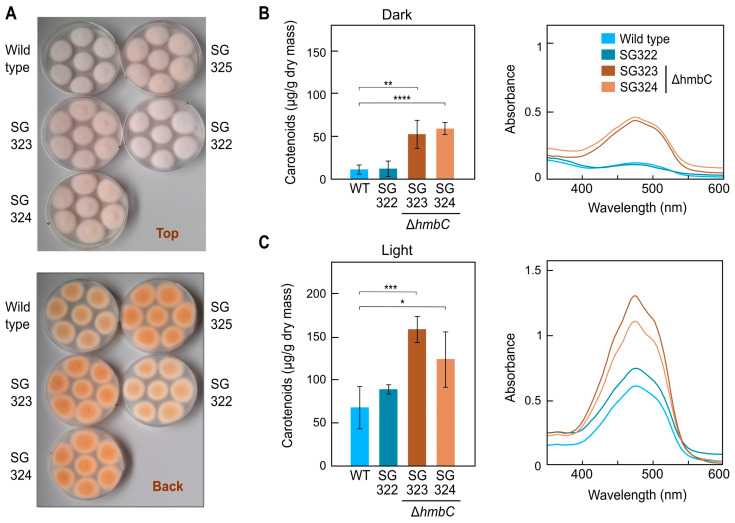
Effect of *hmbC* deletion on carotenoid production. (**A**) Agar cultures of wild type and four transformants grown for 7 days in the dark at 30 °C. (**B**) Content of carotenoids in the wild type (WT), the ectopic transformant SG322, and the Δ*hmbC* transformants SG323 and SG324 grown in the dark. Strains were cultured for 7 days on minimal agar medium. Right graph: Absorption spectra of the acetone extracts used in the carotenoid analysis. (**C**) Results with the same cultures incubated under continuous illumination. Student’s *t* test: * *p* < 0.05, ** *p* < 0.01, *** *p* < 0.001, **** *p* < 0.0001.

The increased orange pigmentation of Δ*hmbC* mutants suggested a higher carotenoid accumulation. To test this, the wild type, the Δ*hmbC* strains SG323 and SG324, and the ectopic transformant SG322, were grown in darkness (Figure 2A) or under light on minimal agar medium, and their carotenoid contents were measured (Figure 2B,C). In accordance with the colonies shown in Supplementary Material, Figure S5C, those of Δ*hmbC* strains SG323, SG324, and SG325 exhibited a more intense color than the colonies of the wild type and the ectopic transformant SG322. As predicted, Δ*hmbC* mutants exhibited increased carotenoid levels in the dark (Figure 2B), with about a 3–4-fold change compared to the control strains. Carotenoid contents were also increased to similar extents in the light in relation to the control strains, but not at the same proportion (Figure 2C). Absorption spectra shapes and wavelengths of the maximal absorbance peaks of their total carotenoid extracts were similar, indicating lack of differences in the accumulated carotenoid mixtures (right panels in Figure 2B,C). These results suggest a possible repressive role of HmbC in the regulation of carotenogenesis in *F. fujikuroi*. 

**Figure 3:**
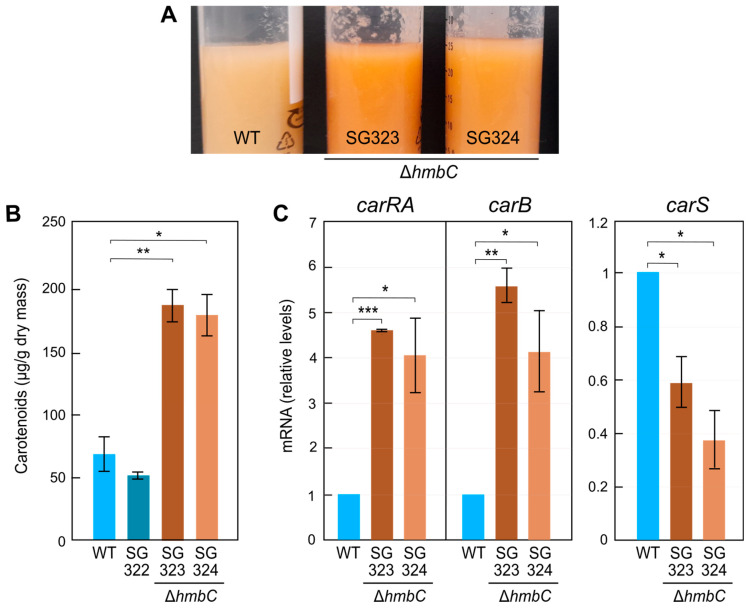
Effect of *hmbC* deletion on carotenoid production and *car* gene expression in submerged conditions. (**A**) Samples of 7-day-old submerged cultures of wild type (WT), and Δ*hmbC* transformants SG323 and SG324 grown in the dark at 30 °C. (**B**) Carotenoid content of the strains under the conditions shown in panel A. The ectopic SG322 transformant was also included in the analysis. (**C**) Transcript levels of the *carRA*, *carB*, and *carS* genes in 3-day-old cultures of the wild type and Δ*hmbC* strains at the same conditions. Only significant differences are indicated according to Student’s *t* test: * *p <* 0.05, ** *p* < 0.01, *** *p* < 0.001.

To determine if the regulatory effect of the *hmbC* deletion on carotenogenesis was carried out at transcription level, RT-PCR analysis of *car* genes was performed from mycelia grown in submerged conditions, used in former *F. fujikuroi* expression studies. First, we confirmed that the difference in pigmentation between wild type and Δ*hmbC* mutants was maintained in liquid cultures in the dark (Figure 3A), in this case, with a three-fold increase in the carotenoid content (Figure 3B). The mRNA levels of *carRA* and *carB*, i.e., the genes for key enzymes of carotenogenesis whose promoters were used in the pull-down assays, were analyzed by RT-PCR in these conditions. Because of the major role of gene *carS* in their regulation, this was also included in the analysis. The results showed a 5–6-fold increase in the amounts of *carRA* and *carB* mRNA in the Δ*hmbC* strains compared to the wild type (Figure 3C), indicating that the deletion of the gene affects, directly or indirectly, *carRA* and *carB* expression. Interestingly, the analysis showed a noticeable decrease in *carS* mRNA levels in the Δ*hmbC* mutants. As CarS is a repressor of carotenogenesis, this could also explain the observed phenotype. 

### 3.6. Other ΔhmbC Phenotypic Alterations

**Figure 4:**
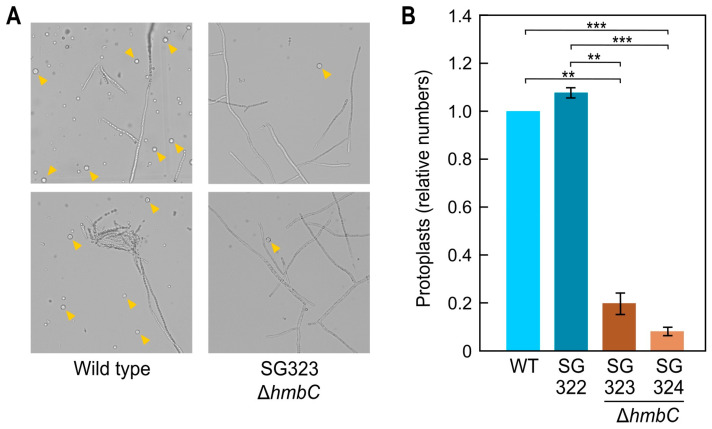
Protoplast formation in the Δ*hmbC* mutants and control strains. (**A**) Representative microscopy images of the production of protoplasts of the wild type and Δ*hmb*C mutant SG323 after 8 h in enzymatic solution. 100 mL of Darken medium were inoculated with a small mycelium plug and incubated for 3 days at 30 °C stirring at 200 rpm. Then, 100 mL of fresh ICI medium was inoculated with 1 mL of the previous culture, and it was incubated overnight at 30 °C. The mycelium was vacuum filtered through sterile filter paper and placed into 25 mL of enzyme solution. Yellow arrowheads indicate visible protoplasts in the photographs. (**B**) Protoplast formation after 8 h. The number of wild type protoplasts was taken as 1. The results show average and standard deviation from two independent experiments. Statistical identifiers of Student’s *t* test: ** *p* < 0.01, *** *p* < 0.001.

In experiments addressed to transform Δ*hmbC* mutants, we noticed a very low protoplast production by these strains (Figure 4A). In two independent transformants, the number of protoplasts decreased approximately 5–10-fold compared to the control strains (Figure 4B). This alteration may reflect possible changes in the cell wall composition of these mutants, indicating pleiotropy of the *hmbC* mutation.

**Figure 5:**
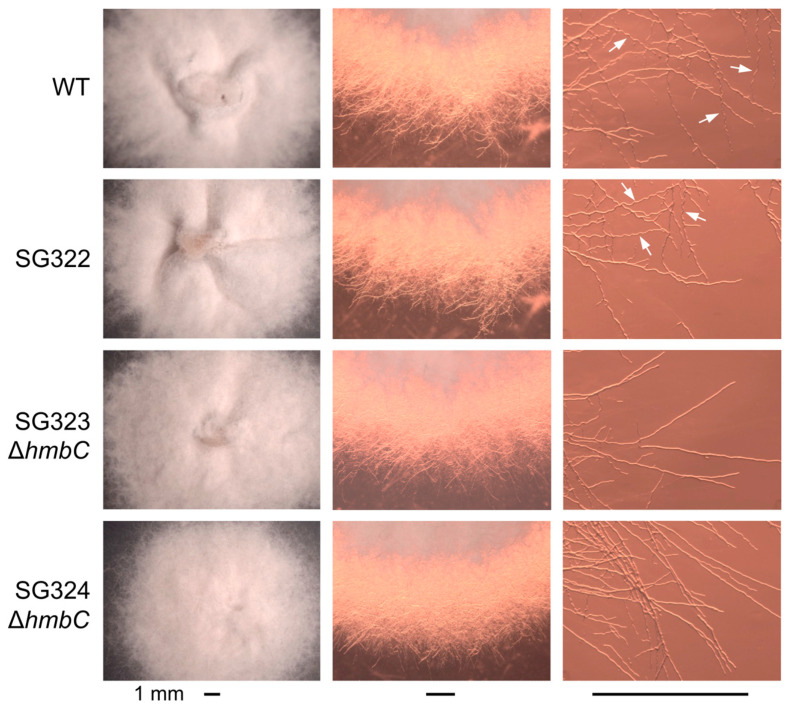
Effect of 1.2 M sorbitol on the morphology of colonies and hyphae at colony borders. Left column: aspect of representative colonies in sorbitol-supplemented medium. Wrinkles on the colony surface are more prominent in the control strains. Central column: differences in the density and appearance of colony edges can also be seen under a stereoscopic microscope with backlighting. Right column: microscope photographs of hyphae at colony edges. Curvy hyphae, indicated by arrows, are less evident in the Δ*hmbC* transformants. The bars below each column correspond to 1 mm.

If the sharp decrease in protoplast production in the mutants is due to changes in the chemical composition of their cell walls, such changes could have consequences for sensitivity to certain chemicals and stress conditions. As a first approach, the effect of osmotic stress was analyzed in the SG323 and SG324 Δ*hmbC* mutants and the two control strains containing the *hmbC* gene. For this, the four strains were grown on minimal medium supplemented with 1.2 M sorbitol or 0.68 M NaCl. Although all strains showed a similar radial growth (Supplementary Material, Figure S7), morphological differences were observed between control strains and deletion mutants in sorbitol-supplemented medium (Figure 5). The colonies of the wild-type and SG322 strain showed a greater tendency to wrinkle on their surface than those of the Δ*hmbC* colonies (left pictures). A more detailed study under stereoscope and microscopy revealed differences in the developing hyphae at the colony edges (central pictures). Under these conditions, the hyphae of the wild-type and ectopic control showed a greater degree of curling than the hyphae of the *hmbC* mutants (right pictures). Growth of the same strains was also investigated on media with either 0.0125% SDS or with different concentrations of calcofluor white or Congo red. No differences were observed in calcofluor-supplemented plates and only minor differences in colony diameter were observed under SDS (Supplementary Material, Figure S8). In the case of the effect of Congo red, preliminary observations suggested a larger colony diameter also in the *hmbC* mutants compared to the wild-type and control strain at 100 µg/µL of this compound, and the differences were not statistically significant. In addition, no differences in colony morphology were observed in the presence of SDS, calcofluor, or Congo red.

## 4. Discussion

The aim of this work was to identify new regulatory proteins involved in the regulation of carotenoid biosynthesis through a biotin-mediated pull-down of proteins capable of binding to *car* gene promoters. The data yielded different candidate proteins that could be worthy of further study. Here, we concentrated our attention on an HMG-box protein presumably able to bind the promoter of the *carB* gene, which we called HmbC after its *A. nidulans* ortholog. In *A. nidulans*, HmbC interacts with the velvet protein VeA, which is involved in the regulation of sexual and asexual development, as well as in secondary metabolism [24]. The role of HmbC in the formation and viability of cleistothecia and ascospores was analyzed in relation to VeA, but the effect of *hmbC* mutation on secondary metabolism in *A. nidulans* remains to be investigated. The deletion of *hmbC* resulted in down-regulation of the *MAT1-1* and *MAT1-2* mating-type genes [24], but the same effect was produced by deletions of other HMG genes, *hmbA* or *hmbB*, suggesting functional redundancy. Connections to the sexual cycle are frequent in HMG proteins. As an outstanding example, deletion of the 12 HMGB genes found in *P. anserina* showed that 11 are involved in different aspects of sexual development [28].

The closest relative of *hmbC* in *S. cerevisiae* is the gene encoding the Hmo1 protein. Deletion of *HMO1* caused severe growth defects, decreased plasmid stability, and changes in chromatin structure, as inferred from increased sensitivity to digestion with micrococcal nucleases [25]. In fact, according to the *Saccharomyces* Genome Database [29], Hmo1p physically or genetically interacts with 290 genes or gene products, indicating widespread cellular functions. Like HmbC, Hmo1p has two globular domains called A box and B box. Box A has low similarity to the consensus HMG box, plays a role as a dimerization domain, and has low affinity for DNA [30,31]. However, this box contributes to DNA bending [32]. In contrast, B box has a more canonical sequence, with higher affinity for DNA but lower structural specificity [30,31], and it participates in DNA-binding affinity but not in DNA bending [32]. Similar functions could be expected for the HmbC-equivalent domains in *F. fujikuroi*, especially for the B domain due to the higher degree of similarity with the Hmo1p equivalent region, but this needs experimental demonstration. The high evolutionary distance between *S. cerevisiae* and *F. fujikuroi* leaves open the possibility of a high degree of functional divergence between both proteins.

Blast analyses found HmbC/Hmo1 orthologs in other fungi, but to our knowledge, there are no published studies on their functions. In *N. crassa*, a global analysis of KO mutants was performed for all its genes [33]. The orthologue of HmbC in this fungus is NCU03126, of 314 residues, and of 61.33% identity with HmbC of *F. fujikuroi*. However, no information is available on the phenotype of the NCU03126 KO mutant. Thus, *hmbC* of *F. fujikuroi* is the third such gene to be investigated in fungi.

The deletion of *hmbC* in *F. fujikuroi* produced an enhanced carotenoid biosynthesis, either on surface cultures, in the dark or under illumination, or in dark-grown submerged cultures. The higher carotenoid content was consistent with the 5–6-fold increase found in the mRNA levels of the *carRA* and *carB* genes in the dark, indicating that the phenotype is caused by a partial upregulation of biosynthetic genes. This can be explained by an increased transcription or a longer half-life of these mRNAs, but a higher transcriptional activity seems the most likely hypothesis, presumably involving a change in chromatin organization. These results agree with the identification of HmbC protein bound to the promoters of the *carB* gene, but not with the lack of binding to the *carRA* promoter region. However, a global effect on chromatin organization could also occur in *carRA* and other adjacent genes through the interaction of HmbC in the *carB* regulatory region. Repressive chromatin structure states may spread from sequence-specific nucleation sites [34] and one of such sites could be the *carB* regulatory sequence. Therefore, HmbC could be a negative regulator acting in the *car* cluster on this site.

An unexpected result, which adds uncertainty to the cause of the upregulation of the *carRA* and *carB* genes, is that the deletion of *hmbC* produces a decrease of approximately 50% in the mRNA levels of the *carS* gene. We do not have information on proteins capable of binding to the *carS* promoter, but this result suggests that HmbC also participates in *carS* expression, possibly influencing the organization of chromatin structure at the *carS* region. It has been previously described that CarS is a negative regulator of *carRA* and *carB* mRNA levels and that the expression of these genes is very sensitive to *carS* transcript levels. The expression of *carS*, whose mRNA levels are relatively low, is finely tuned in *F. fujikuroi* to allow adequate carotenoid synthesis in response to light. Thus, the increased expression of *carS* leads to a lack of induction of the *car* genes by light and, consequently, to an albino phenotype [19]. Therefore, the decrease in the expression of the *carS* gene could explain the observed effect on *carRA* and *carB* transcript levels and, consequently, on carotenoids synthesis.

Considering the general mechanisms of chromatin rearrangement in which HMGB proteins participate [6], and their ability to bind to nonspecific DNA sequences [4,5], it is to be expected that the HmbC protein of *F. fujikuroi* is not specifically involved in carotenogenesis and is related to other cellular processes. In this sense, and in line with what was observed with the *hmbC* mutation in *A. nidulans*, other phenotypic alterations were found in *hmbC* mutants, such as a lower efficiency in protoplast formation. Since the generation of protoplasts relies on the degradation of cell wall by specific lytic enzymes, this trait indicates alterations in the chemical composition of the cell wall in the *hmbC* mutants. These presumed changes are possibly related to other observations in the mutants, such as alterations in hyphal development patterns under osmotic stress conditions. These phenotypic changes are not particularly striking, and, in fact, under normal growth conditions, no morphological differences were observed in the development of their colonies. Similarly, the mutants of the *hmbC* orthologue in *A. nidulans* show no apparent differences in development and growth [24]. The functional relevance of HmbC in both species is less than that observed with its orthologue Hmo1p in *S. cerevisiae*, the deletion of which results in severe growth impairment and reduced colony size [35]. The lesser effect of *hmbC* mutations in *F. fujikuroi* and *A. nidulans* may be due to a greater functional redundancy of their HMG proteins. Hmo1 participates in the synthesis and biogenesis of ribosomes and *HMO1* promoter is regulated by TOR [36,37]. In response to nutrient limitations or DNA damage, the ribosome synthesis is repressed, while it is induced when nutrients are available.

Future attention should be also dedicated to the putative HMG protein CCT62089, also found in our pull-down screening. This is the closer homolog in *F. fujikuroi* of HmbB of *A. nidulans*, which plays a role in ascospore and conidia viability, and has also functions in hyphal morphology, sensitivity to oxidative stress, and metabolite production [38]. However, in contrast to HmbC, this protein is preferentially located in the mitochondria and is also involved in maintenance of redox homeostasis [39] and integrity of mitochondrial genome [38].

## 5. Conclusions

This work added a new piece to the regulatory network that controls carotenogenesis in *F. fujikuroi*. Its discovery was based on a scrutiny of proteins capable of binding to the promoters of *car* genes, thus inferring its possible regulatory connection with CarS as a protein that controls the expression of these genes. With the available information, we do not know whether HmbC participates in the regulation of carotenogenesis directly through its structural genes, or indirectly by participating in the control of the expression of its negative regulator CarS. However, an action at both levels cannot be ruled out. In *S. cerevisae*, transcription-promoting actions have been described for Hmo1p, such as its participation in nucleosome-free chromatin stabilization [40], or in start-site selection by RNA polymerase II through its interaction with TFIID [26]. Activities of this type by HmbC could explain the downregulation of *carS* gene expression resulting from *hmbC* deletion. However, chromatin-assembly-promoting activity has also been described for Hmo1p in the same yeast [27], and such an action could explain the increased transcription of the *carRA* and *carB* genes in the *hmbC* mutant. Anyhow, the information available is still very limited and this is just a tentative hypothesis. Further investigation is needed to unravel the mechanism of action of HmbC in *F. fujikuroi* carotenogenesis, as well as the possible participation of other proteins of the same family, including HmbB.

## Figures and Tables

**Table 1:** Combined counts of individual peptides for pull-downs with promoters of *carB* and *carRA*/*carX* incubated with nuclear extract and full protein extract of wild type (WT) and *carS* mutant SG39 (S). The number of individual peptides accumulated, and proteins identified in the negative control were subtracted. The proteins are arbitrarily ordered according to the number of times identified from the wild-type extracts and pulled down with *carB* promoter (P*carB*). Those with a putative counterpart in the *S. cerevisiae* proteome are indicated.

			P*carB*		P*carRA/X*	
Protein	Yeast Ortholog	Features	WT	S	WT	S
CCT62089		uncharacterized protein	62	108	41	12
CCT66893		related to NAD+ ADP-ribosyltransferase	40	86		
CCT62893	Hmo1p	related to nonhistone chromosomal protein	34	58		
CCT71516	Hho1p	related to HHO1-histone H1 protein	20	39	21	3
CCT72522		related to Pas7p	14	64	3	
CCT62055	Rpl36ap	probable 60S ribosomal protein L36	12	13		
CCT70243	Yku80p	related to transcriptional regulator ATRX homolog	10	36	2	
CCT61955	Apn1p	probable AP endonuclease	8	57		
CCT67445	Htb1p	probable HTB1-histone H2B	8	12		
CCT68541	Rfa1p	probable single-stranded DNA-binding protein	7	54		
CCT66187	Rim1p	related to single-stranded DNA-binding protein	6	14	3	
CCT67540	Gbp2p	related to Gbp2p	6	14		
CCT69891	Mgm101p	Probable mitochondrial genome maintenance protein	5	30	2	
CCT73695		uncharacterized protein	5	18	6	6
CCT63952		related to excision repair protein RAD4	2	28		
CCT66006	Top1p	probable TOP1-DNA topoisomerase I	2	18		
CCT73736		related to Glu/Asp-tRNA amidotransferase subunit		20		

**Table 2:** *F. fujikuroi* genes whose proteins contain HMG domains. They are ordered according to their protein number. Proteins identified in the pull-down study are shown in bold.

Gene	Protein	Residues	Features
*FFUJ_01566*	CCT61934	512	related to mating type 1–2 protein
*FFUJ_14410*	**CCT62089**	310	uncharacterized protein
*FFUJ_00897*	CCT62544	102	probable NHP6B-nonhistone chromosomal protein
*FFUJ_00508*	**CCT62893**	354	related to nonhistone chromosomal protein
*FFUJ_02729*	CCT65758	540	related to nonhistone chromosomal protein
*FFUJ_02997*	CCT66004	1085	related to VPS33-vacuolar sorting protein
*FFUJ_03111*	CCT66114	269	uncharacterized protein
*FFUJ_13386*	CCT67192	672	uncharacterized protein
*FFUJ_13684*	CCT67469	698	uncharacterized protein
*FFUJ_05685*	CCT69772	223	mating type protein (Mat1-2-1)

## Data Availability

Not applicable.

## Supplementary Materials

The following supporting information can be downloaded at: https://www.mdpi.com/article/10.3390/genes14081661/s1. Table S1. Primers used for the generation of the constructs, probe and verification of DNA integration in the transformants. Table S2. Primers used for quantitative RT-PCR. Table S3. Sequences of primers used for amplification of car promoters. Table S4. PCR setups and programs used to amplify biotin-labelled promoters. Figure S1. Construction of pMFL11 plasmid. Figure S2. (A) Genomic organization of the genes of the carotenoid pathway in *F. fujikuroi*. (B) Simplified scheme of the *F. fujikuroi* carotenoid pathway. (C) Amplification of biotin-coupled promoter regions of P*carB* and P*carRA*/*carX*. (D) Control steps of the streptavidin pulldown confirming the binding of biotinylated P*carB* (bait) to the streptavidin beads. Figure S3. Cladogram representation of a neighbour-joining phylogenetic tree without distance corrections of the 10 proteins of the HMG-box family in *F. fujikuroi*. Figure S4. (A) Clustal alignment between the HmbC protein of *F. fujikuroi* (CCT62893) and (A) HmbC of *A. nidulans* (CBF89496) and (B) Hmo1 of *S. cerevisiae* (KAJ1540853). Figure S5. Screening of possible *hmbC* deletion transformants by PCR. (A) Map of the genomic *hmbC* region showing the expected homologous recombination generating the deletion. (B) Electrophoresis of PCR carried out with genomic DNA of wild type and nine transformants using primer set PS6 to test the integration of the HygR cassette in the *hmbC* locus by homologous recombination. (C) Aspect of the pigmentation of 4-day old colonies of the wild type and the positive transformants in panel B. (D) Electrophoresis of PCR amplifications with genomic DNA of wild type and the eight transformants using PS7 primer set that bind to the surrounding of the *hmbC* locus, that are absent in the deletion cassette. Figure S6. Identification by Southern blot of transformants with *hmbC* deletion. (A) Restriction map of the genomic *hmbC* region showing the expected homologous recombination generating the deletion. (B,C), Southern blot analysis of genomic DNA from the wild type and transformants 1, 17, 31, and 34 digested with *Eco*RI or *Pst*I. Figure S7. Effect of osmotic stress in the *hmbC* mutants and control strains. Figure S8. Effect of SDS on growth of Δ*hmbC* mutants and control strains. (A) Agar cultures of the indicated strains for 7 days on DG minimal medium and 0.013% SDS stress medium at 30 °C under dark conditions. (B) Size of the colonies after 7 days on DG minimal medium and 0.013% SDS medium at 30 °C.
