# Supplementary material for: HmbC, a Protein of the HMG Family, Participates in the Regulation of Carotenoid Biosynthesis in Fusarium fujikuroi
Source: Genes (Basel). 2023 Aug 21;14(8):1661. doi: 10.3390/genes14081661 (PMC10454146; doi:10.3390/genes14081661)
Supplement: Supplementary file 1 [file genes-14-01661-s001.zip › genes-2533446-supplementary.pdf]

HmbC, a protein of the HMG family, participates in the regulation of carotenoid biosynthesis in *Fusarium fujikuroi*

Marta Franco-Losilla, Steffen Nordzieke, Ingo Feldmann, M. Carmen Limón, and Javier Avalos

SUPPLEMENTARY MATERIAL

Table S1. Primers used for the generation of the constructs, probe and verification of DNA integration in the transformants.

Set	Primer	Sequence [5′ to 3′]	Use
PS1	HMGc_5_1F	GTAACGCCAGGGTTTTCCCAGTCACGACG ACCAGTCAGCGCTGCCCGCG	PCR *hmbC* deletion cassette
	HMGc_3_2R	GCGGATAACAAGTTCACACAGGAAACAG CCCACATCAAAAGCAAAGGCC	
PS2	HMGc_3_2F	CTCCTTCAATATCATCTTCTGTCTCCGACG CTTTCTTTACTCTCCGTTG	PCR 3′ fragment of *hmbC*
	HMGc_3_2R	GCGGATAACAAGTTCACACAGGAAACAG CCCACATCAAAAGCAAAGGCC	
PS3	HMGc_5_1F	GTAACGCCAGGGTTTTCCCAGTCACGACG ACCAGTCAGCGCTGCCCGCG	PCR 5′ fragment of *hmbC*
	HMGc_5_1R	ATCCACTTAACGTTACTGAAATCTCCAAC CGCTGACGACTTGATGGGACGCGT	
PS4	HPH-6F	GTCGGAGACAGAAGATGATATTGAAG GAGC	PCR Hyg^R cassette
	HPH-6R	GTTGGAGATTTCAGTAACGTTAAGTGGAT	
PS5	HMGc_5_1F	GTAACGCCAGGGTTTTCCCAGTCACGACG ACCAGTCAGCGCTGCCCGCG	PCR 5′ region of *hmbC*-Hyg^R cassette
	HPH-6R	GTTGGAGATTTCAGTAACGTTAAGTGGAT	
PS6	hmgbC_5_2F	GCAGCCAACACCTGTAGCAT	PCR test Δ*hmbC*
	Hph-11R	TGAGCTGATGCTTTGGGCCGA	
PS7	HMGc_5_1F	GTAACGCCAGGGTTTTCCCAGTCACGACG ACCAGTCAGCGCTGCCCGCG	PCR test Δ*hmbC*
	HMGc_5_1R	ATCCACTTAACGTTACTGAAATCTCCAAC CGCTGACGACTTGATGGGACGCGT	
PS8	hmgbC_3_2F	GCATGATACCTAAGGAGAGT	Southern probe
	hmgbC_3_2R	GAACTCCGTGAGCAATACAG	

Table S2. Primers used for quantitative RT-PCR

Gene	Primers	Sequence [5´ to 3´]
carS	RtFfcarS-1F	GATACCCGGCGGAAAGGTTA
	RtFfcarS-1R	CTGACAGTCCATTTCAGCGC
carB	RTcarB-1F	TCGGTGTCGAGTACCGTCTCT
	RTcarB-1R	TGCCTTGCCGGTTGCTT
carRA	RTcarRA-1F	CAGAAGCTGTTCCCGAAGACA
	RTcarRA 1R	TGCGATGCCCATTTCTTGA
β-tub	Tub-2F	CCGGTGCTGGAAACAACTG
	Tub-2R	CGAGGACCTGGTCGACAAGT
gpdA	RT-gpdh-1F	GTGACCTCAAGGGCGTTCTG
	RT-gpdh-1R	CGAAGATGGAGGAGTTGGTGTT

Table S3. Sequences of primers used for amplification of *car* promoters

Name	Target promoter	Sequence [5′ to 3′]	Use
PcarB_fw_biotin	P*carB*	**Biotin**-TGGGGTGAAGCGTG GAGGATG	Amplification of *carB* promoter
PcarB_bw	P*carB*	TTTGGCTGTAAAAAGTGAA GATGCTCAGTG	
PcarRAcarX_fw_biotin	P*carRA/carX*	**Biotin**-CATGATGATATGTG GACTATGATATTAC	Amplification of double *carRA/carX* promoter
PcarRAcarX_bw	P*carRA/carX*	CATTTTGACAAAGATTTAT TTCGTG	

Table S4. PCR setups and programs used to amplify biotin-labelled promoters

**P*carB***	**P*carRAcarX***	**µl**	**MM (x15)**	**Program**			
10x BioTaq		10	150	**P*carB***		**P*carRAcarX***	
$MgCl_2$		3	45	94°C-2′		94°C-2′	
PcarB_fw_biotin	PcarB_bw	4	60	94°C-30′′		94°C-30′′	
PcarRAcarX_fw_biotin	PcarRAcarX_bw	4	60	55°C-30′′	x35	52°C-30′′	x34
dNTPs		2	30	72°C-28′′		72°C-55′′	
template: *F. fujikuroi* WT genomic DNA		2	30	72°C-10′		72°C-10′	
BioTaq		1	15	4°C-hold		4°C-hold	
H_2O		74	1,110				
Total		100	1,500				

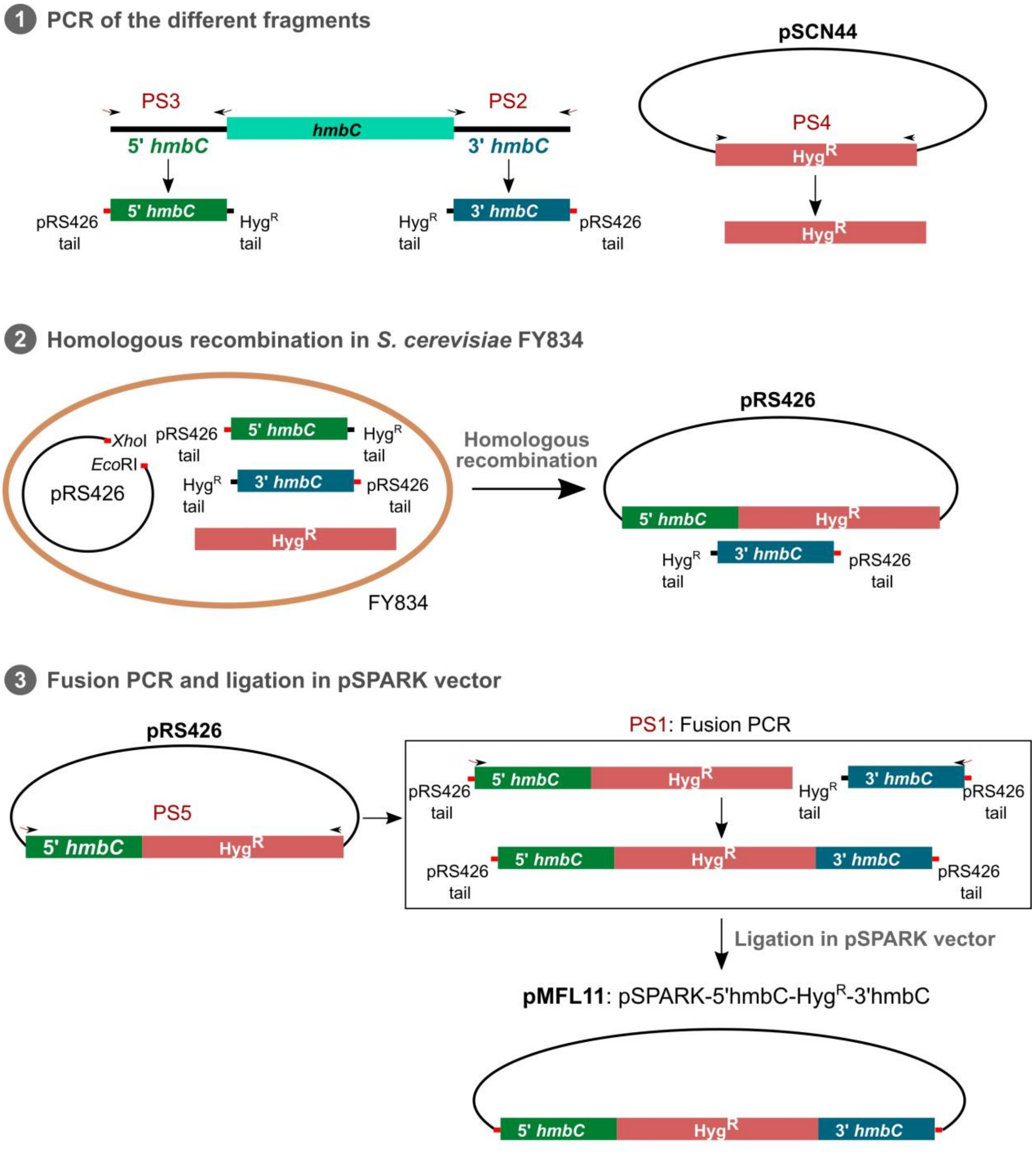

Figure S1. Construction of pMFL11 plasmid. Steps 1, 2 and 3 are described in the section of material and methods. Primer sets (PS#) are indicated in red. Primer locations are indicated by small arrows.

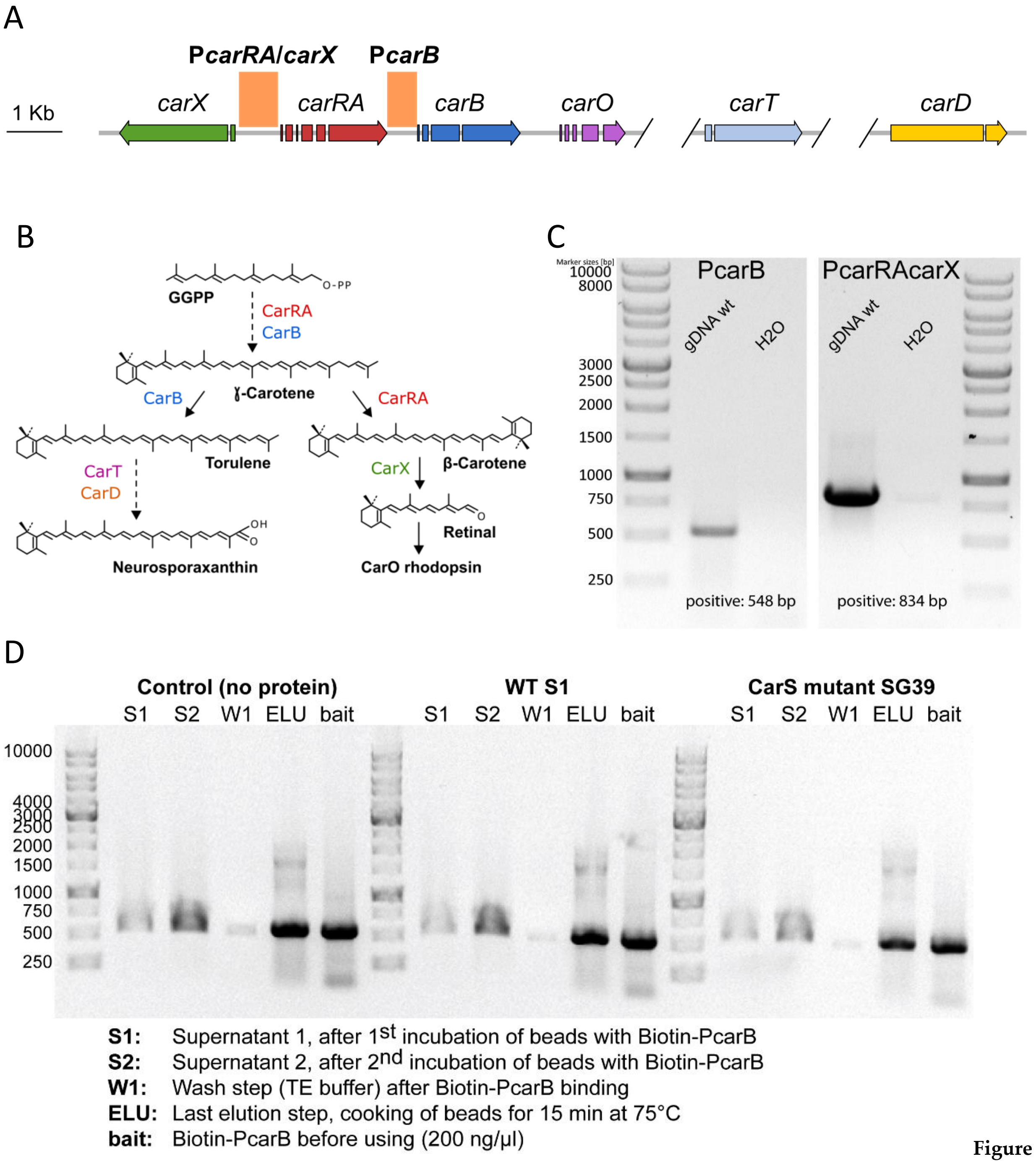

Figure S2. (**A**) Genomic organization of the genes of the carotenoid pathway in *F. fujikuroi*. The *carRA*, *carB*, and *carX* genes are clustered with the *carO* rhodopsin gene. The regulatory segments P*carRA*/*carX* and P*carB* used in the pulldown assay are indicated. Genes *carT* and *carD* are at other genomic locations. Gaps in the genes indicate introns. (**B**) Simplified scheme of the *F. fujikuroi* carotenoid pathway. The steps in which each enzyme is involved is indicated. Dashed lines represent several enzymatic steps. (**C**) Amplification of biotin-coupled promoter regions of P*carB* and P*carRA*/*carX*. gDNA wt: amplification from genomic DNA of the wild type. (**D**) Control steps of the streptavidin pulldown confirming the binding of biotinylated P*carB* (bait) to the streptavidin beads. Beads were incubated twice with biotinylated P*carB* and supernatant was analyzed (S1 and S2). Additionally, a washing step with TE buffer (W1) and the final eluate (ELU) were visualized.

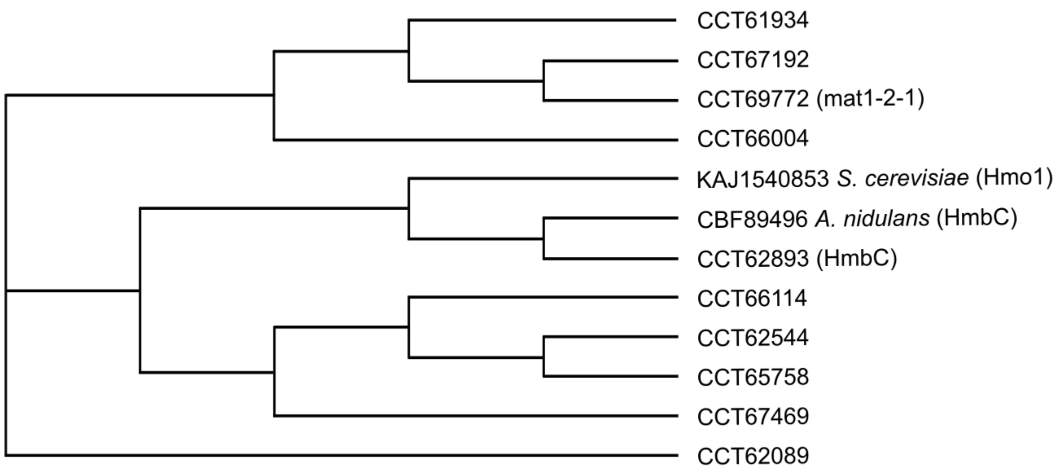

Figure S3. Cladogram representation of a neighbour-joining phylogenetic tree without distance corrections of the 10 proteins of the HMG-box family in *F. fujikuroi*. The sequences of the presumed HmbC orthologs in *S. cerevisiae* and *A. nidulans* are also included.

A

```
CCT62893   MARPKKQVVEKQQQQQQPAPVPIPPQHIQQYPQHPQPIAAVPQPAMPMPMPPPVGVPQPQ 60
CBF89496   ---------------------------------------------------------MPK 3
                                                                     *:

CCT62893   AVMPQRVVEADSFLRVRDSAVGRLTTILELLRSFTADYVRQTNLLLGEPTAEGSQDNLLA 120
CBF89496   NDSKTVTIDVEEFTKTRDSFLAKLAQLQSLTFELSRAYINHTSAVLGQDNANVDISAITN 63
                 .::.:.* :.*** :.:*: : .*  .::  *:.:*. :**: .*: . . :

CCT62893   NFEHAAQ--QLIMPIPELAPPVEEKKERKKRTIDPNAPKRPLTPYFLYMQHARSIIANDL 178
CBF89496   TLAASLRDTGVLAAAGTGSGAESGEKKKRKRRADPNAPKRTLTPFFLYMHHNRARIAEEL 123
           .:  : :   ::      :   . :*:::**  ******* ***:****:* *: **::*

CCT62893   GSEAPKGAVQEEGQRRWAHMGPQEKQGWNNAYQYNLRLYNARVHSYKHGNPLAKNMTDEE 238
CBF89496   GPDAKPKDVSNEGTRRWAEMPDSQKEVWKKLYADNLAAYREKVAAYKAGLPFDRDDNDKA 183
           * :*    *.:** ****.*  .:*: *:: *  **  *. :* :** * *: :: .*:

CCT62893   ALKYAEDFNVPMSEIKDAAQNEAPADDQDAIAEQLQAVAAAPAIDEPEQAETPAKTPKKA 298
CBF89496   ADQLHLDVA--AAEASDEEEEE----EEDEHGEE--------EEEEEEESPEPAKEPTPP 229
           * :   *.    :* .*  ::*    ::*  .*:          :* *::  *** *.

CCT62893   TGGRKRKTATPAA----AAEEAKPAPASPDKKRRRTSTKAAAAEPQEEPKKSGRKKT--- 351
CBF89496   PPKRRRSEGKPSKDVSSPVVEKKGRNESPEKKRRGAA-----KKDKEEPRKSLGGESKRS 284
              *:*. ..*:      . * *    **:**** ::      : :***:**   ::

CCT62893   ----KSS------ 354
CBF89496   KKKRKSDVGGDDE 297
               **.
```

16 % identities (15 / 94)

49 % identities (36 / 73)

Total: 87 identities

B

```
CCT62893   MARPKKQVVEKQQQQQQPAPVPIPPQHIQQYPQHPQPIAAVPQPAMPMPMPPPVGVPQPQ 60
KAJ1540853 -----------------------------------------------MTTDPSVKLKSAK 13
                                                          *   * * : . :

CCT62893   AVMPQRVVEADSFLRVRDSAVGRLTTILELLRSFTADYV-----------RQTNLLLGEP 109
KAJ1540853 DSLV------SSLFELSKAANQTASSIVDFYNAIGDDEEEKIEAFTTLTESLQTLTSGVN 67
             :       .*::.: .:*    ::*::: .::  *                .*  *

CCT62893   TAEGSQDNLLANFEHAAQQLIMPIPELAPPVEEKKERKKRTIDPNAPKRPLTPYFLYMQH 169
KAJ1540853 HLHGISSELVNPIDDDKDAI------IAAP--VKAVRRKIERDPNAPKKPLTVFFAYSAY 119
             .* ..:*:  ::.  : :      :* *   *  *:*   ******:*** :* *  :

CCT62893   ARSIIANDLGSE----APKGAVQEEGQRRWAHMGPQEKQGWNNAYQYNLRLYNARVHSYK 225
KAJ1540853 VRQELREDRQKAGLPPLSSTEITQEISKKWKELSDNEKEKWKQAYNVELENYQREKSKYL 179
           .*. : :*   .       .  : :* .::* .:. :**: *::**: :*. *: .  .*

CCT62893   HGNPLAKNMTDEEALKYAEDFNVPMSEIKDAAQNEAPADDQDAIAEQLQAVAAAPAIDEP 285
KAJ1540853 EAKKNGT---------------LPPASL-----ENGPTHAPVPIPFSLQHAAEPPVEKRP 219
           ..:  ..               :* :.:     ::.*:.    *  .** .*  *. ..*

CCT62893   EQAETPAKTPKKATGGRKRKTATPAAAAEEAKPAPASPDKKRRRTSTKAAAAEPQEEPKK 345
KAJ1540853 HDDDGSSEKKKKKKKK----------------------DKKKDKSNSSI----------- 246
           .: :  ::. ** .                        ***: ::.:.

CCT62893   SGRKKTKSS 354
KAJ1540853 --------- 246
```

14 % identities (13 / 94)

30 % identities (22 / 73)

Total: 52 identities

Figure S4. (A) Clustal alignment between the HmbC protein of *F. fujikuroi* (CCT62893) and **(A)** HmbC of *A. nidulans* (CBF89496) and **(B)** Hmo1 of *S. cerevisiae* (KAJ1540853). Partial identities in the A (orange) and B (green) domains as well as total found in the pairwise comparisons are indicated.

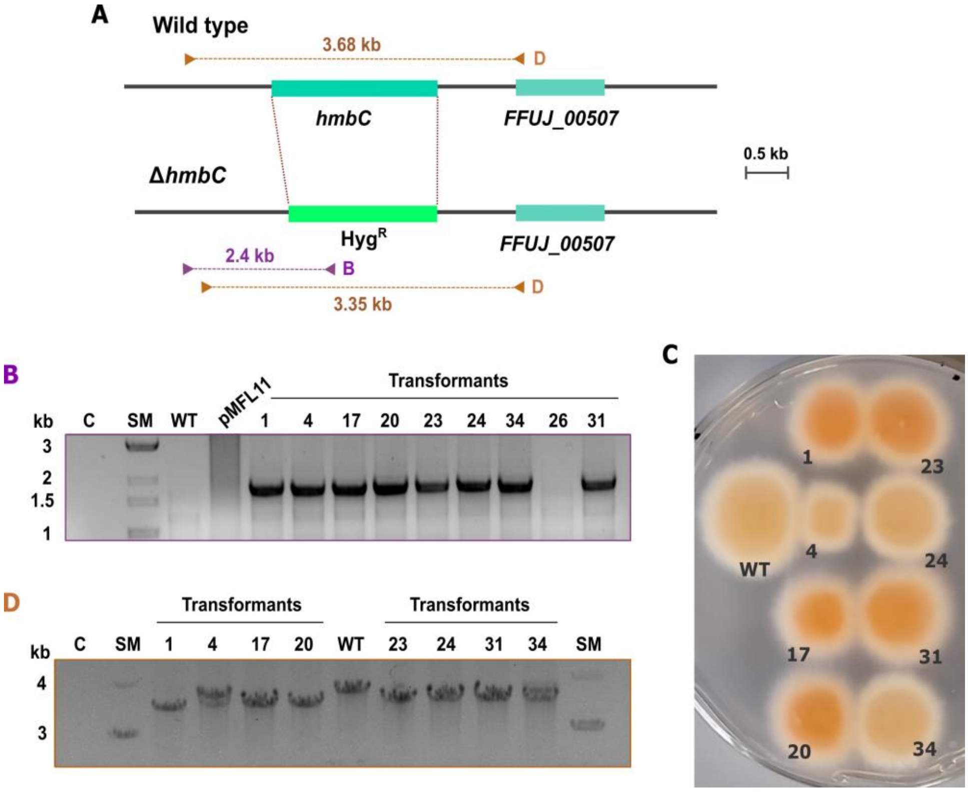

Figure S5. Screening of possible *hmbC* deletion transformants by PCR. **(A)** Map of the genomic *hmbC* region showing the expected homologous recombination generating the deletion. Expected amplicons of the PCR shown in panels B and D are indicated. **(B)** Electrophoresis of PCR carried out with genomic DNA of wild type and nine transformants using primer set PS6 to test the integration of the Hyg^R cassette in the *hmbC* locus by homologous recombination. The positive 2.4 kb band was observed in eight of the transformants. As expected, no amplification was obtained from the wild type and from pMFL11 plasmid. **(C)** Aspect of the pigmentation of 4-day old colonies of the wild type (WT) and the positive transformants in panel B. Five of them (1, 17, 20, 23 and 31) exhibit a deeper pigmentation. **(D)** Electrophoresis of PCR amplifications with genomic DNA of wild type and the eight transformants using PS7 primer set that bind to the surrounding of the *hmbC* locus, that are absent in the deletion cassette. The replacement should result in a 0.3 kb reduction in the size of the PCR product. Except transformant 4, the other transformants exhibit the expected shift in the product size. C: control without DNA. SM: size markers.

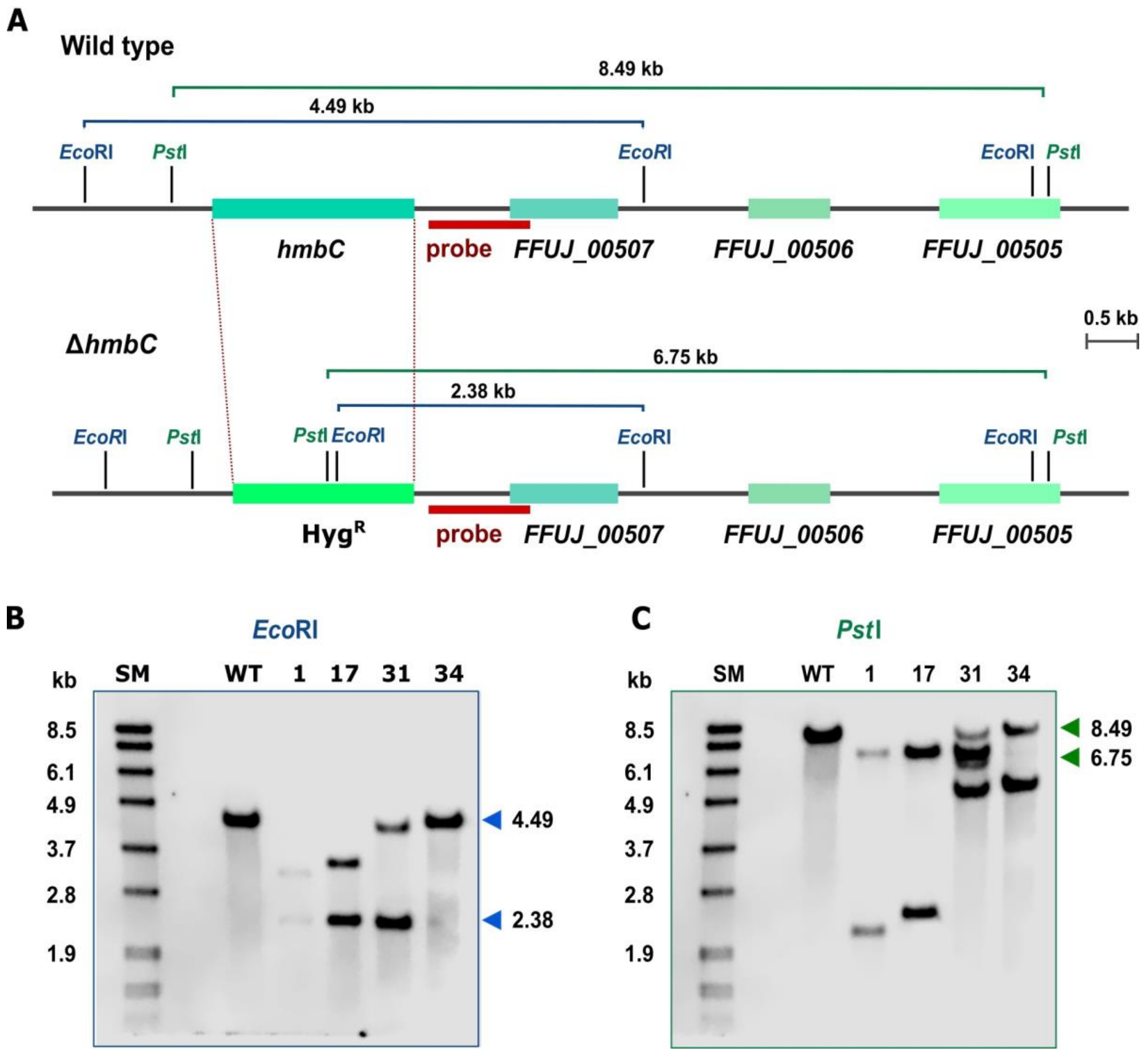

Figure S6. Identification by Southern blot of transformants with *hmbC* deletion. **(A)** Restriction map of the genomic *hmbC* region showing the expected homologous recombination generating the deletion. A probe obtained by PCR using PS8 primer set is shown as well as the expected hybridizing bands upon *Eco*RI or *Pst*I digestions. (**B)** and **(C),** Southern blot analysis of genomic DNA from the wild type and transformants 1, 17, 31, and 34 digested with *Eco*RI or *Pst*I. SM: Size markers. Sizes of relevant bands are indicated on the right.

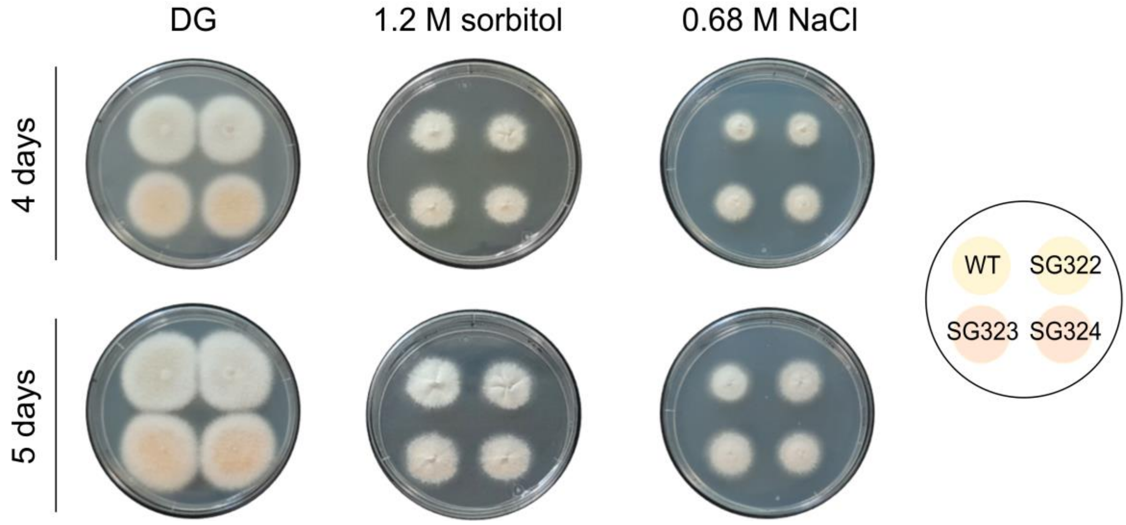

Figure S7. Effect of osmotic stress in the *hmbC* mutants and control strains. A small sample of mycelium from the wild type and *hmbC* deletions strain were inoculated on different osmotic stress media with tweezers from a fresh colony. These strains were grown for 4 and 5 days at 30 °C under dark conditions in DGasn minimal medium, without or with 1.2 M sorbitol or 0.68 M NaCl.

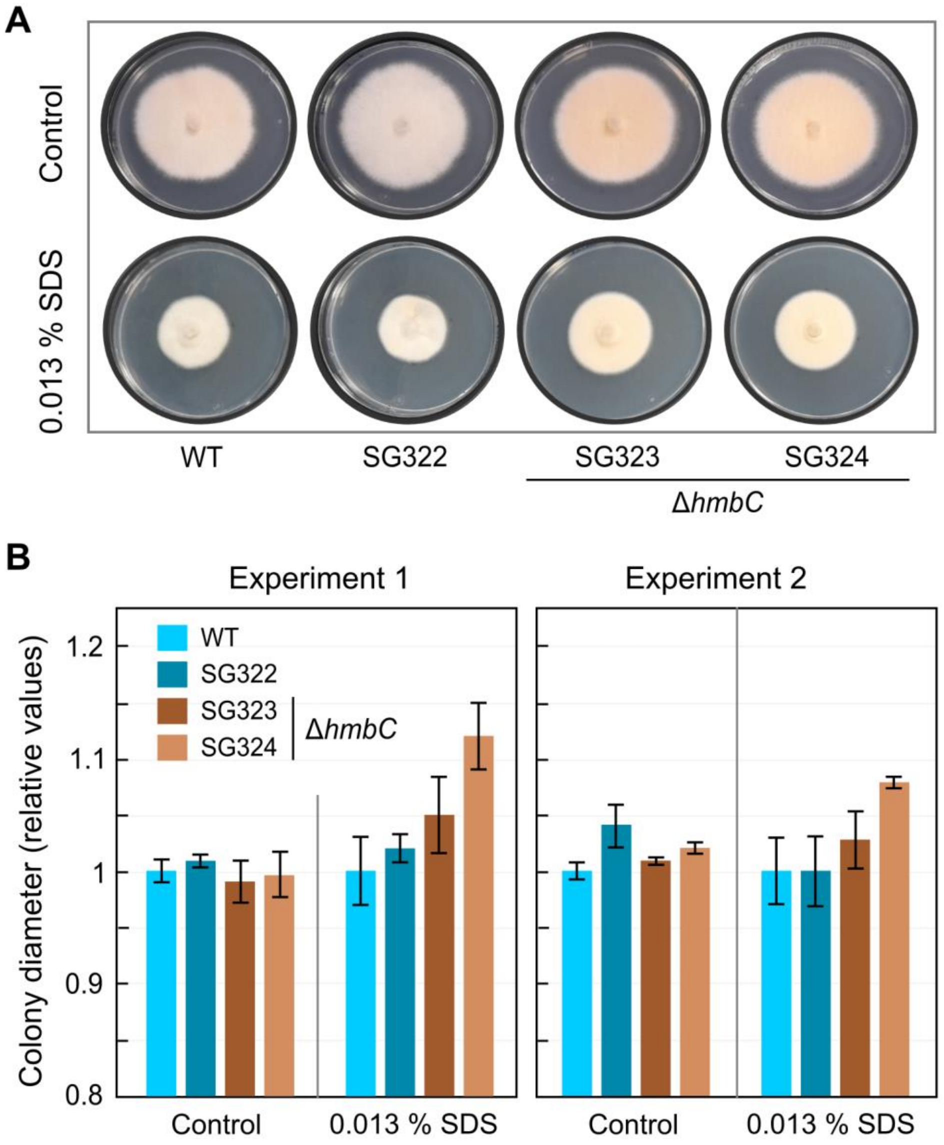

Figure S8. Effect of SDS on growth of Δ*hmbC* mutants and control strains. **(A)** Agar cultures of the indicated strains for 7 days on DG minimal medium and 0.013% SDS stress medium at 30 °C under dark conditions. **(B)** Size of the colonies after 7 days on DG minimal medium and 0.013% SDS medium at 30 °C. Data show the average and standard error from two inde*p*endent experiments with four parallel cultures in Petri dishes. The statistic Student's *t* test gave values of $p > 0.05$, indicating no significant differences among the strains in the presence of SDS.
